# Environmental filtering rather than dispersal limitation dominated plant community assembly in the Zoige Plateau

**DOI:** 10.1002/ece3.9117

**Published:** 2022-07-11

**Authors:** Jianping Yang, Peixi Su, Zijuan Zhou, Rui Shi, Xinjing Ding

**Affiliations:** ^1^ Key Laboratory of Land Surface Process and Climate Change in Cold and Arid Regions Northwest Institute of Eco‐Environment and Resources, CAS Lanzhou China; ^2^ University of Chinese Academy of Sciences Beijing China

**Keywords:** community assembly, distance decay, environmental filtering, species assemblage similarity

## Abstract

Identifying the mechanisms that underlie the assembly of plant communities is critical to the conservation of terrestrial biodiversity. However, it is seldom measured or quantified how much deterministic versus stochastic processes contribute to community assembly in alpine meadows. Here, we measured the decay in community similarity with spatial and environmental distance in the Zoige Plateau. Furthermore, we used redundancy analysis (RDA) to divide the variations in the relative abundance of plant families into four components to assess the effects of environmental and spatial. Species assemblage similarity liner declined with geographical distance (*p* < .001, *R* ^2^ = .6388), and it decreased significantly with increasing distance of total phosphorus (TP), alkali‐hydrolyzable nitrogen (AN), available potassium (AK), nitrate nitrogen (NO_3_^+^–N), and ammonia nitrogen (NH_4_^+^–N). Environmental and spatial variables jointly explained a large proportion (55.2%) of the variation in the relative abundance of plant families. Environmental variables accounted for 13.1% of the total variation, whereas spatial variables accounted for 11.4%, perhaps due to the pronounced abiotic gradients in the alpine areas. Our study highlights the mechanism of plant community assembly in the alpine ecosystem, where environmental filtering plays a more important role than dispersal limitation. In addition, a reasonably controlled abundance of Compositae (the family with the highest niche breadth and large niche overlap value with Gramineae and Cyperaceae) was expected to maintain sustainable development in pastoral production. These results suggest that management measures should be developed with the goal of improving or maintaining suitable local environmental conditions.

## Graphical abstract

Environmental filtering dominated plant community assembly in the Zoige Plateau.

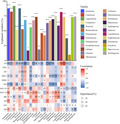

## 1 INTRODUCTION

In ecology, determining the mechanisms that produce species diversity within communities is an important goal (McGill, 2010). A major viewpoint in explaining community assembly mechanisms is deterministic processes (e.g., species interactions and environmental filtering) based on niche differentiation (Keddy, 1992). According to this theory, niche separation occurs along environmental or temporal niche axes (Silvertown, 2004) due to variations in species' capacity to cope with environmental filtering (Cadotte & Tucker, 2017) or limiting similarity (Violle et al., 2011). The stochastic processes of neutral theory (e.g., random death, dispersal, and speciation) have also been proposed as an important viewpoint in explaining species distribution patterns. The importance of stochastic vs. deterministic processes in community assembly is under continuous debate (HilleRisLambers et al., 2012; Hubbell, 2011). In some cases, neutral theory has been able to effectively predict species abundance, suggesting that functional differences between species are not necessary to explain the observed patterns of biodiversity in nature. However, some studies suggested that most species did not present random death, dispersal, and speciation (Chave, 2004; Purves & Turnbull, 2010); moreover, other studies have shown that community biodiversity cannot be understood without considering deterministic mechanisms such as environmental filtering (Cadotte & Tucker, 2017; Laliberté, Zemunik, et al., 2014) or species interactions (Maire et al., 2012; Zuppinger‐Dingley et al., 2014). The roles of deterministic and stochastic processes in community assembly may differ depending on the horizontal scales studied. For example, as horizontal scales shrank, environmental heterogeneity decreased, resulting in lower habitat preferences and thus a greater contribution of stochastic processes than deterministic processes (Chase, 2014). In terms of integration, an increasing number of researchers are attempting to combine neutral and niche theories by incorporating neutral theory drift into niche theory or niche theory into the neutral theory framework (Hubbell, 2006; Matthews & Whittaker, 2014; Pinto & MacDougall, 2010; Stokes & Archer, 2010).

Environmental filtering is a deterministic process in the absolute sense that abiotic factors prohibit organisms lacking specific physiological characteristics from surviving in local populations (Kraft et al., 2015). Dispersal limitation cannot be necessarily identified as stochastic processes, which can be deterministic, stochastic, or both (Zhou & Ning, 2017). It has been reported that the pervasive pattern of plant distributions in natural communities has been mainly attributed to the combined effect of environmental filtering and dispersal limitation. Environmental filtering or dispersal limitation has been observed to increase or decrease with tree life stages, respectively (Yang et al., 2016). Moreover, the effects of environmental filtering vs. dispersal limitation on community assembly can differ based on the taxa involved (Franklin et al., 2013; Padial et al., 2014). Environment filtering, for example, plays a greater role in shaping the community assembly of temperate deciduous broad‐leaved forests in China (Liu et al., 2015). Conversely, non‐random dispersal may be a major driver of early successional riparian vegetation zonation and biodiversity (Franklin et al., 2013). We can evaluate these impacts by dividing the contribution of different factors to community assembly.

Community similarity decay is common in many taxa and regions and can be caused either by dispersal limitation or by spatial configuration. The use of distance decay models is crucial to understanding how biological assemblages vary across space and identifying the processes that drive these variations (Peguero et al., 2022; Soininen et al., 2007). Changes in environmental conditions between sites are typically associated with increasing geographic distances, and relying solely on geographic distances to infer dispersal limitations may confuse the effect of ecological filtering with that of dispersal limitation (Gilbert & Lechowicz, 2004). With this aim, several studies have assessed the relative explanatory power of environmental and spatial factors for particular biological groups, as dispersal limitation is predicted to produce higher explanations of community similarity with spatial than with environmental variables, while species sorting via ecological niches is predicted to produce higher explanations with environmental than with spatial variables (Legendre et al., 2009; Shi et al., 2021).

Alpine areas are zones of high species endemism. Indeed, because of the substantial abiotic gradients in alpine regions, a niche‐based explanation of alpine plant distribution appears to be particularly evident (Körner & Kèorner, 1999). On the contrary, dispersal limitation was an important factor for alpine plant community diversity (Klanderud & Totland, 2007), which was inherently linked to the dispersal capability of species (Vellend et al., 2007), but may also be influenced by other factors (water and climate shifts with elevation, competition, herbivory, mutualists, etc.). Recently, many factors have resulted in the loss of diversity in numerous alpine plant species. Such poisonous weed expansion, which causes alpine meadow ecosystem unbalance, is one of the considerable ecological problems and an important index of alpine meadow degeneration (Liu & Diamond, 2005; Zhao et al., 2010). The majority of studies on variables influencing plant community assembly are carried in temperate or warm areas, while studies in alpine ecosystems being rare. As a result, from the viewpoint of protecting alpine biodiversity, researching the variables influencing species distributions may allow predictions of future range changes and control of poisonous weed expansion in alpine plant communities.

We assessed (i) how soil variables affected each plant family's relative abundance, (ii) how the decay of community similarity with spatial distance and environmental distance, and (iii) which plant families of poisonous weeds mainly threaten palatable grass (Cyperaceae and Gramineae) for livestock. We studied the soil physicochemical properties related to species distribution patterns (Laliberté, Legendre, et al., 2014; Laliberté, Zemunik, et al., 2014) and the relative abundance of each plant family. Our first hypothesis is that alpine plant community assembly is affected by dispersal limitation to some degree. Our second hypothesis is that the variation of species assemblages explained by environmental filtering should be higher than spatial variables. We expect that the results will contribute to a better understanding of plant community assembly mechanisms influencing the maintenance and generation of plant biodiversity in the alpine ecosystem.

## 2 MATERIALS AND METHODS

### 2.1 Study sites

The study was conducted in the alpine meadow area of the Zoige Plateau (33°10′ to 34°06′N, 101°36′ to 103°25′E), in the northeastern corner of the Qinghai–Tibet Plateau (Figure S1). This region has a typical humid and semihumid continental monsoon climate of the plateau cold temperate zone (Bai et al., 2013). There is more rain and heat in the same season, and the air temperature swings greatly from day to night. The annual mean air temperature ranges between 0.6 and 1.2°C, with a long frost season. The annual mean precipitation is 600–800 mm (Ding et al., 2021), while the annual evaporation is higher compared with the annual mean precipitation.

### 2.2 Species distribution data

In April 2018, we selected 6 permanent meadows (s1‐s6) along gradients of altitude in Gamaliang, ranging from 3500 to 4000 m asl. At each site, eight plots with the same altitude but different geographic locations were randomly selected to conduct species distribution and environmental data (Figure S1), and the geographic coordinates were recorded in Appendix S2. In July 2020, we recorded all plant species on three 2 m × 2 m quadrats and measured the relative abundance (the number of individuals of one species as compared to that of the whole community) of every species. In Appendix S2, species were given full Latin names and their relative abundance was shown. A total of 79 species belonging to 25 families were recorded.

### 2.3 Environmental and spatial variables

We studied the soil physicochemical properties as soil physicochemical properties influence species diversity and distribution patterns (Laliberté, Zemunik, et al., 2014). At the sampling locations, nine soil cores were collected at 30 cm radial distances from the ground. At each plot, composite soil samples were made by mixing three soil cores. In total, 144 samples were obtained. Soil samples were dried in a darkened area at room temperature. The soil was crushed and filtered through a 2‐mm mesh sieve after contaminants were removed. Physical and chemical characteristics were then measured using these samples. A pH meter was used to measure soil pH in a 1:2.5 (mass: volume) soil water suspension. The potassium dichromate volumetric technique was used to determine organic matter (SOM). The high‐temperature combustion method was used to determine total organic carbon (TOC). The semimicroKjeldahl method was used to determine total nitrogen (TN). Nitrate nitrogen (NO_3_^+^–N) and ammonia nitrogen (NH_4_^+^–N) were determined by Nessler's reagent spectrophotometry. Flame photometry was used to measure available potassium (AK) in 1 M ammonium acetate extracts (FP640, INASA). 0.5 M NaHCO_3_ was used to extract available phosphorus (AP), which was then measured using the molybdenum blue method. The total phosphorus (TP) was also determined using the molybdenum blue method. Soil moisture content (SWC) and soil density (BK) were determined using the core cutter method (Bao, 2000).

Furthermore, the spatial eigenfunctions were generated using principal coordinates of neighbor matrices (PCNM) from the geographic coordinates of 48 quadrats, and the spatial variables were represented by sections of the spatial eigenfunctions with positive eigenvalues (Legendre et al., 2009).

### 2.4 Statistical analysis

#### 2.4.1 Species assemblage similarity between altitude groups and within altitude groups

The Bray–Curtis dissimilarity is extensively used to assess the dissimilarity of species assemblages between two different locations (Ricotta & Podani, 2017). The Bray–Curtis dissimilarity algorithm is as follows: (Legendre & Legendre, 2012):

$$BC_{\mathrm{ij}}=1–\frac{2C_{\mathrm{ij}}}{s_{i}+s_{j}}$$

where *C*_*ij*_ is the sum of the lower values for just the species shared by both locations. The total number of specimens counted at both sites is represented by *S*_*i*_ and *S*_*j*_.

Species assemblage similarity was calculated as 1 − dissimilarity of the Bray–Curtis metric. Analysis of similarity (Anosim) and permutational multivariate analysis of variance (Adonis) are non‐parametric statistical tests widely used in the field of ecology (Assis et al., 2015; Myers & Harms, 2011). Anosim was performed in the vegan package to determine whether species assemblage dissimilarities between altitude groups were greater than within altitude groups, and Adonis was used to identify whether species assemblage dissimilarities contained significant differences between altitude groups (Oksanen et al., 2015). The analysis of beta diversity (variation in community structure) over several spatial or temporal scales was facilitated by permutation multivariate dispersion (PERMDISP) (Anderson, 2014). The mean distance to a group centroid was utilized to describe β‐diversity for each group, and PERMDISP was used to assess β‐diversity differences between altitude groups (Price et al., 2019). Functional diversity of different altitude groups was extracted using FD package (Laliberté, Legendre, et al., 2014).

#### 2.4.2 Correlation analysis and multiple linear regression model

Correlation analysis (Spearman correlation) and multiple linear regression models together with variation partitioning analysis were used to clearly analyze the impact of numerous soil factors on each family's relative abundance. A Pearson correlation analysis was performed by the cor.test function in the stats package (Field et al., 2012). To identify the main soil factors, a multiple regression model (lm function in the stats package) with variation decomposition analysis (calc.relimp function in the relaimpo package) was used (Grömping, 2015).

#### 2.4.3 Distance decay analysis

Geographical distances were extracted using the distHaversine function of the Geosphere package (Hijmans et al., 2017), and they were calculated from the geographic coordinates among pairs of sampling points. Environmental distances were calculated as the Euclidean distance among soil variables. The distance decay model was run to test whether species assemblage similarity (based on the Bray–Curtis similarity from abundance) decreased as geographical or environmental distances increased, and it was performed by standard and partial Mantel tests, using the vegan package (Oksanen et al., 2013). DDRs were calculated as the slopes of linear least‐squares regression.

#### 2.4.4 Variation partitioning

The ordiR2step function in the vegan package was used to choose environmental variables by forwarding selection. If the significance threshold (*p* > .05) was achieved, or if there was no improvement in the selection criterion (*R* ^2^) after adding any new factors, the test was terminated. The selection procedure retained 3 PCNM and 8 soil variables (Tables S2, S3 in Appendix S1). Redundancy analysis (RDA) was performed using the rda function in the vegan package to divide the variation in a species assemblage (Yeh et al., 2015). The variation in species assemblage was divided into four parts based on variables that remained after selection: (a) variation that is purely explained by environmental variables; (b) variation that is jointly explained by environmental variables and spatial variables; (c) variation that is purely explained by spatial variables; and (d) variation that is unexplained (Shi et al., 2021).

#### 2.4.5 Neutral community model (NCM)

The variation in species assemblages explained only by spatial variables was not fully attributable to dispersal limitation (Legendre et al., 2009), so we built two neutral community models for A1 (3500–3700 m altitude gradient) and A2 (3800–4000‐m altitude gradient) to estimate the dispersal limitations of the plant community. The estimated migration rate (*m*) was employed in this model to assess the dispersal limitation, and higher *m* values imply less dispersal limitation (Burns et al., 2016).

#### 2.4.6 Niche breadth and niche overlap indexes of different plant families

The niche.width function of the spaa package was used to calculate niche breadth indexes of 25 plant families, and niche overlap indexes among 25 plant families were run by the niche.overlap function of the spaa package (Levins, 1968). According to the niche breadth index, the species were divided into three categories: generalist species, specialist species, and neutral taxa (Wilson & Hayek, 2015). All analyses were performed in R 3.6.0 (R Core Team, 2013).

## 3 RESULTS

### 3.1 Species assemblage similarity and environmental drivers of relative abundance

The Anosim analysis revealed species assemblage dissimilarities between altitude groups were greater than those within altitude groups (*R* ^2^ = 1, *p* = .001) (Figure S2). Furthermore, Adonis and Anosim analysis revealed a significant difference in species assemblage between random altitude groups (Table S1 in Appendix S1).

**FIGURE 1:**
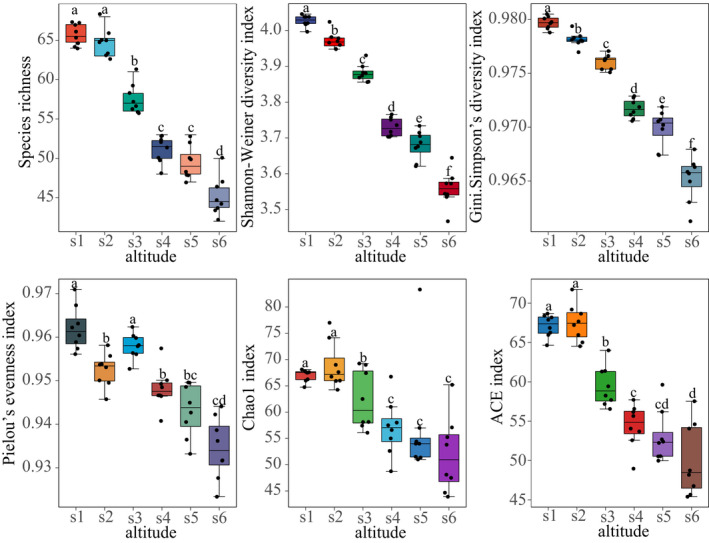
Species richness, diversity, and functional diversity of plant community at various altitudes. Bars marked with the same letter showed no significant difference (*p* > .05) between the two altitude groups

The species richness, Simpson's diversity index, Shannon–Wiener diversity index, Pielou's evenness index (except s3), Chao 1 index, and ACE index of the plant community decreased with increasing altitude (Figure 1).

**FIGURE 2:**
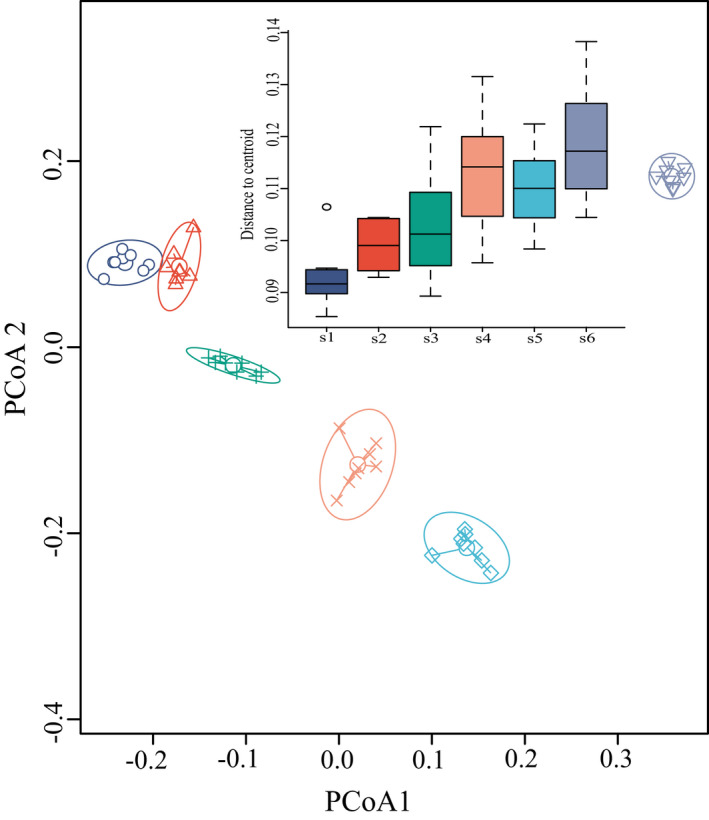
β‐diversity differences between altitude groups based on PERMDISP analysis. PERMDISP, permutation multivariate dispersion; the β‐diversity of each group was characterized by its distance to the centroid

The PERMDISP analysis showed significant β‐diversity differences between altitude groups (*p* = .001), and it showed that the β‐diversity of different altitude groups was mainly separated from each other in the first axis (Figure 2). Distances to the centroid of the altitude groups increased with altitude.

**FIGURE 3:**
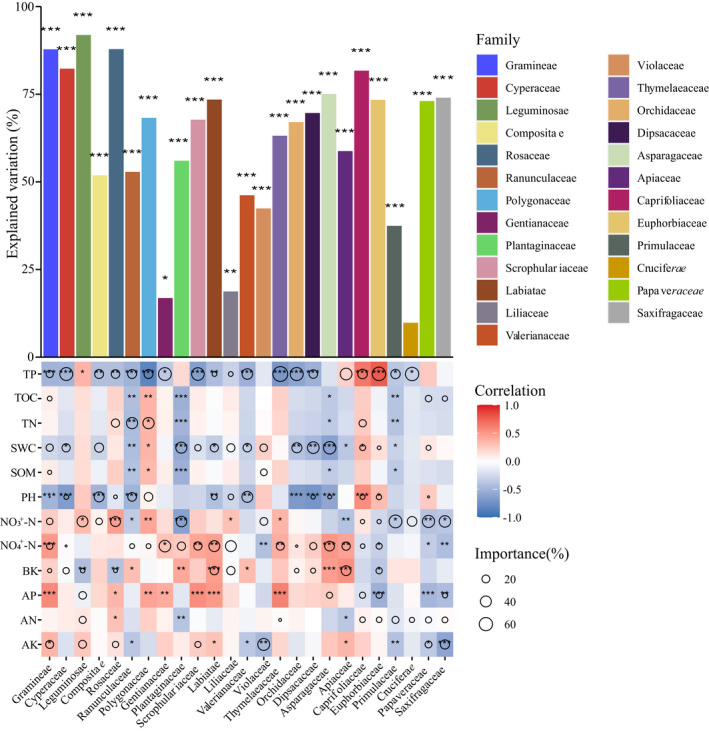
Contribution of various soil variables to the relative abundance of each species based on correlation and the best multiple regression model. The Spearman correlations between soil variables and relative abundance were shown by the colors. The size of the circle reflected the variation explained rate of the selected variable. The significance of relative abundance explained by soil variables was indicated by the asterisks above the bar (*p < .05, **p < .01, ***p < .001). AK, soil available potassium; AN, alkali‐hydrolyzable nitrogen; AP, soil available phosphorus; BK, soil density; NH_4_^+^–N, soil ammonia nitrogen; NO_3_^+^–N, soil nitrate nitrogen; pH, pH of soil in 1:2.5 (mass:volume); SOM, soil organic matter; SWC, soil moisture content; TN, soil total nitrogen; TOC, soil total organic carbon; TP, soil total phosphorus

The relative abundance of most plant families was strongly predicted by TP, NO_3_^+^–N, NH_4_^+^–N, and SWC. For example, the relative abundance of Cyperaceae, Scrophulariaceae, Thymelaeaceae, and Orchidaceae was substantially linked to TP. NO_3_^+^–N was essential to Rosaceae and Plantaginaceae relative abundance, while NH_4_^+^–N was important to Labiatae and Asparagaceae relative abundance. SWC influenced the relative abundance of Plantaginaceae and Asparagaceae (Figure 3).

### 3.2 Distance decay of species assemblage similarity

**FIGURE 4:**
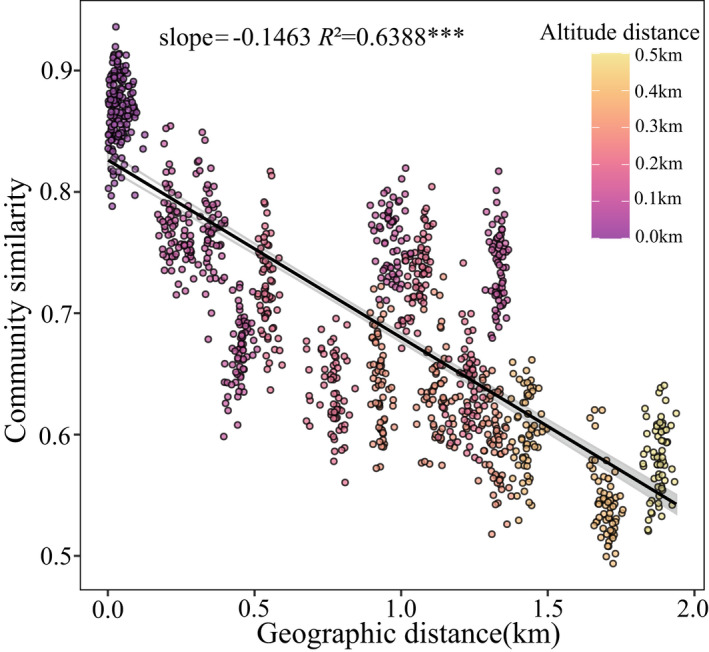
Decay of community similarity with geographical distance is based on Bray–Curtis similarity. Linear least‐squares regression was shown by solid lines. Significant decay was indicated with asterisks (***p < .001)

**FIGURE 5:**
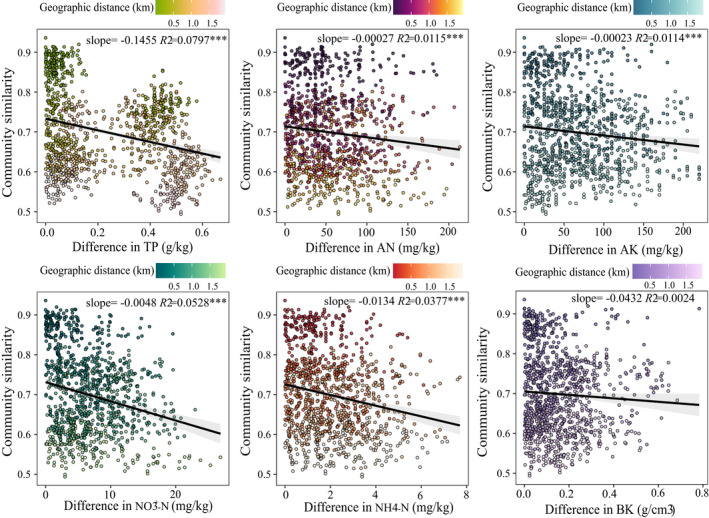
Decay of community similarity with environmental distances is performed by Mantel tests. The significance of decays was indicated by the asterisks (*p < .05, **p < .01, ***p < .001). AK, soil available potassium; AN, alkali‐hydrolyzable nitrogen; BK, soil density; NH_4_^+^–N, soil ammonia nitrogen; NO_3_^+^–N, soil nitrate nitrogen; TP, soil total phosphorus

The DDRs were significant negative values (*p* < .001), and the fitness values were relatively high (*R* ^2^ = .6388), showing that species assemblage similarity declined rapidly with geographical distance, and it decreased with increasing altitude distance (Figure 4). The Mantel tests revealed that species assemblage similarity significantly decreased with increasing distance of TP, AN, AK, NO_3_^+^–N, and NH_4_^+^–N, and it decreased with increasing geographical distance (Figure 5). TP and NH_4_^+^–N distance mainly affected species assemblage similarity with a slope of −0.1455 and −0.0134, respectively. Differences in AK minimally affected species assemblage similarity with a slope of −0.00023. The distances of NO_3_^+^–N and AN affected species assemblage similarity modestly, with slopes of −0.0048 and −0.00027, respectively (Figure 5).

### 3.3 Variation partitioning in species assemblage

**FIGURE 6:**
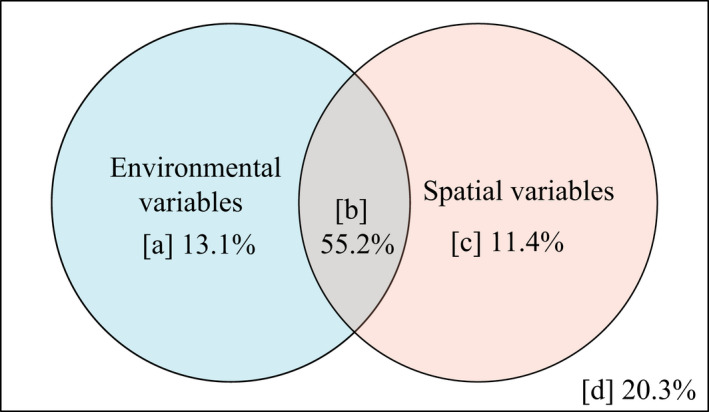
Variation partitioning in species assemblages is explained by environmental and spatial variables. (a) Variation that is purely explained by environmental variables; (b) variation that is jointly explained by environmental variables and spatial variables; (c) variation that is purely explained by spatial variables; and (d) variation that is unexplained

The results of variation partitioning showed that environmental and spatial factors differed in their effects to explain variation in species assemblage similarity. The majority of the variation in species assemblage similarity was explained by the combined effect of environmental and spatial variables. The contribution of spatial variables, however, was less than that of environmental variables (Figure 6).

**FIGURE 7:**
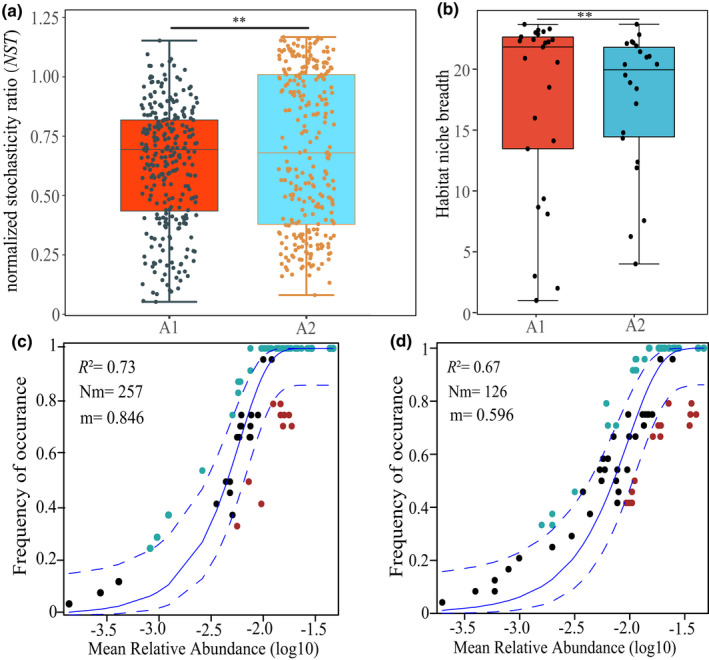
Fitting of the neutral model in two plant communities. (a) NST (normalized stochasticity ratio) of A1 (3500–3700 m altitude gradient) and A2 (3800–4000‐m altitude gradient). (b) A1 and A2 habitat niche breadth. (c) The fit of the neutral model in A1 plant communities. (d) The fit of the neutral model in A2 plant communities. The blue dashed lines indicated the 95% confidence intervals around the model prediction. The fit to the neutral model and the predicted migration rate were shown by *R* ^2^ and *m* values, respectively, *Nm* indicates the metacommunity size times immigration. Significant differences were indicated with asterisks (**p < .01)

The plant communities fit the neutral community model better in the low‐altitude gradient (3500–3700 m) than in the high‐altitude gradient (3800–4000 m). The rate of migration followed a similar pattern (Figure 7). Low‐altitude group had a substantially greater m value than that of high‐altitude group (*p* < .01). The breadth of habitat niches in low‐altitude communities was considerably greater than in high‐altitude groups (*p* < .01, Figure 7).

### 3.4 Niche breadths and overlaps in plant families

In terms of habitat niche breadth at the community level, the Ranunculaceae family had the greatest value in habitat niche breadth, followed by the Cyperaceae and Compositae families, respectively. The Asparagaceae family had the lowest value of habitat niche breadth (Table S4). For Gramineae and Cyperaceae families, Compositae was the family with the highest niche breadth and a large niche overlap value with them (Table S4, Figure S3).

## 4 DISCUSSION

### 4.1 Dominant environmental factors influencing the environment filtering

Despite the fact that specific soil plant communities have been observed and described (Meyer & García‐Moya, 1989), there is absolutely little information accessible on the procedures followed at their community assembly. The soil factor can operate as a powerful abiotic filter, selecting which plants from a given species pool can establish themselves in a given location under edaphically constrained conditions (Weiher et al., 2004). In this study, the relative abundance of various plant families was affected by different soil variables (Figure 3), and the community similarity of different sampling points was between 0.5 and 1 (Figure 5). The findings demonstrated that soil filtering did not result in an all‐or‐nothing response in plant community assembly, similar to a previous study that found soil filtering played a probabilistic role in plant community assembly (Luzuriaga et al., 2015).

In this study, total phosphorus (TP) was the strongest soil filter in the plant community assembly. The alkali‐hydrolyzable nitrogen (AN), available potassium (AK), nitrate nitrogen (NO_3_^+^–N), and ammonia nitrogen (NH_4_^+^–N) played a certain filtering role in the plant community assembly (Figure 5). Previous studies reported that soil heterogeneity increased plant diversity (Baer et al., 2020). Soil heterogeneity has been found to determine the assembly of plant communities (Trepanier et al., 2021; Williams & Houseman, 2014). This is partly consistent with the conclusion of this study that soil variable distance reduced the similarity of species assemblages.

### 4.2 Effect of environmental and spatial variables on community assembly

Basically, spatial structures are generated by two mechanisms (Fortin & Dale, 2009). First, the spatial structures identified in species assembly may be attributed to environmental variation via species‐habitat associations (Legendre et al., 2009). Second, spatial structures may arise from species assemblages, notably through dispersion limitation, which can form aggregated patterns through neutral mechanisms, given that individuals of all species have the same set of demographic rates (Borda‐de‐Água et al., 2007), resulting in spatial autocorrelation of species data. In the present study, we observed a relatively strong (*p* < .001, *R* ^2^ = .6388) distance decay of similarity with geographic distance (Figure 4), suggesting that the spatial structures of plant communities were apparent. We further examined variation partitioning in community assembly. Previous research has found that environmental variables are frequently spatially autocorrelated and have spatial structures (Legendre, 1993), supporting our finding that environmental and spatial factors jointly accounted for a substantial percentage (~60%) of the variation in community composition (Figure 6). This section was further segmented into pure environmental variation ([*a*]), pure spatial variation ([*c*]), and a mix of the two ([*b*]). It is hardly surprising that most of the environmental variation is spatially structured, given the large altitudinal gradient of the sampling points. It is worth noting that environmental composition ([*a* + *c*]) accounted for a sizable proportion of the variation in community composition (66.6% in Figure 6). Our second hypothesis was confirmed by the results. The component ([*c*]) represented the contributions of unobserved factors that are not related to soil but are spatially structured, such as climate characteristics (e.g., directional temperature reduction), or other habitat and biological factors.

It is worth noting that about 20% of the variation was unexplained ([*d*] in Figure 6).

Several hypotheses for the unexplained variation may be given, additional nonspatially structured biological or environmental factors that were not observed in the research might be used to account for this variation. Another reasonable explanation is that it was caused by stochastic processes. The latter hypothesis was theoretically related to the neutral theory. Dispersion has spatial structure and may cause variations in components ([*c*]) and ([*d*]), whereas the effect of drift should manifest in component ([*d*]). If other environmental (e.g., climatic conditions) or biological (e.g., species interactions) variables were assessed and included in the research, the variation decomposition shown in Figure 6 would most certainly alter (Antonelli et al., 2018).

In conclusion, our findings indicated that both deterministic (soil and other spatially structured environmental factors) and stochastic processes were major drivers in the alpine meadow community assembly. Our study results were consistent with the finding that environmental filtering was more important than dispersal limitation in the community assembly of China's temperate deciduous broad‐leaved forests (Liu et al., 2015), and it was consistent with finding that environmental filtering played a larger role in shaping the species composition in a European grassland (Horn et al., 2015), and it was consistent with the result that environmental variables contributed more to community assembly than spatial variables (Shi et al., 2021). Pure spatial variation may correspond to unmeasured environmental variables and neutral processes (Legendre et al., 2009); it is currently impossible to distinguish between these two sources of variation. The neutral model's estimated migration rate (Figure 6) and linear distance decay of species assemblage similarity with geographic distance (Figure 4) revealed that dispersal limitation accounted for a part of the pure spatial variation. The results matched our first hypothesis. Dispersal limitation is inherently linked to the dispersal capability of species (Vellend et al., 2007), but may also be influenced by other factors (water and climate shifts with elevation, competition, herbivory, mutualists, etc.). It is currently impossible to distinguish which factors have limited the dispersal of species.

### 4.3 Limitations and management implications

It is crucial to understand the relative contributions of environmental filtering and dispersal filtering in order to design effective conservation and restoration strategies. When environmental filtering is dominating, actions must be taken to improve or maintain suitable local environmental conditions (Kareksela et al., 2015; Lamers et al., 2015). When dispersal limitation dominates, however, it is critical to protect neighboring source populations and secure dispersal routes (Brederveld et al., 2011; Fraaije et al., 2015). According to our results, the proportion of environmental filtering explained was more than that of spatial variables. Management measures should be developed with the goal of improving or maintaining suitable local environmental conditions.

Biodiversity is an important subject in ecology and society since a significant loss of biodiversity might diminish ecosystem functioning and benefits (Cardinale et al., 2012). High plant diversity can increase ecosystem multifunctionality and resilience to climate change (Delgado‐Baquerizo et al., 2017). The most common ecosystem type on the Zoige Plateau is the alpine meadow. Poisonous weed expansion, which causes alpine meadow ecosystem unbalance, is one of the considerable ecological problems and an important indicator of alpine meadow degeneration (Liu & Diamond, 2005; Zhao et al., 2010). Conservation efforts should prioritize maintaining ecosystem stability and limiting the spread of poisonous weeds in alpine meadows. Cyperaceae and Gramineae belong to palatable families for cattle and sheep. In our current study, the Compositae was the family with the highest niche breadth and large niche overlap value, along with Gramineae and Cyperaceae (Table S4, Figure S3). A reasonably controlled abundance of Compositae is expected to maintain sustainable development in pastoral production.

However, because we did not analyze other environmental (e.g., climatic factors) or biological (e.g., species interactions) variables in our observed research, we cannot confirm or count out their possible impact on community assembly. Furthermore, because our research was conducted in a regional climatic gradient with a humid and semihumid continental monsoon climate, further research along broader climatic gradients with more aridity levels would be required to fully assess the impacts of environmental variables, spatial variables, and species interactions on community assembly.

## 5 CONCLUSION

This study presented evidence supporting the notion that dispersal limitation and environmental filtering jointly governed plant community assembly and that the plant community assembly in alpine meadows was more constrained by environmental filtering than by dispersal limitation. The results highlight that improving or maintaining proper local environmental conditions could maintain and improve the diversity of alpine plants. In addition, a reasonably controlled abundance of Compositae (the family with the highest niche breadth and large niche overlap value with Gramineae and Cyperaceae) was expected to maintain sustainable development in pastoral production. Given the importance of plant community assembly for ecosystem stability and multifunctionality in alpine meadows, future research could focus on the effects of climatic factors and biotic interactions on community assembly.

## AUTHOR CONTRIBUTIONS

**Jianping Yang:** Conceptualization (lead); investigation (lead); methodology (lead); software (lead); visualization (lead); writing – original draft (lead); writing – review and editing (lead). **Peixi Su:** Conceptualization (lead); investigation (lead); methodology (lead); project administration (lead); software (equal); visualization (equal); writing – original draft (lead); writing – review and editing (lead). **Zijuan Zhou:** Investigation (equal); software (equal); visualization (equal); writing – review and editing (equal). **Rui Shi:** Investigation (equal); visualization (equal); writing – review and editing (equal). **Xingjing Ding:** Investigation (equal); validation (equal); visualization (equal); writing – review and editing (equal).

## CONFLICT OF INTEREST

The authors state that the research was conducted in the absence of any commercial or financial relationships that could be construed as a potential conflict of interest.

## Supporting information


Appendix S1

Appendix S2

## Data Availability

Data and R code for correlation and best multiple regression model and variation partitioning are available via figshare (https://figshare.com/s/3cfdafa41aa2acec24a6).
