# Supplementary material for: Environmental filtering rather than dispersal limitation dominated plant community assembly in the Zoige Plateau
Source: Ecol Evol. 2022 Jul 11;12(7):e9117. doi: 10.1002/ece3.9117 (PMC9272205; doi:10.1002/ece3.9117)
Supplement: Supplementary file 1 — Appendix S1 [file ECE3-12-e9117-s001.docx]

**
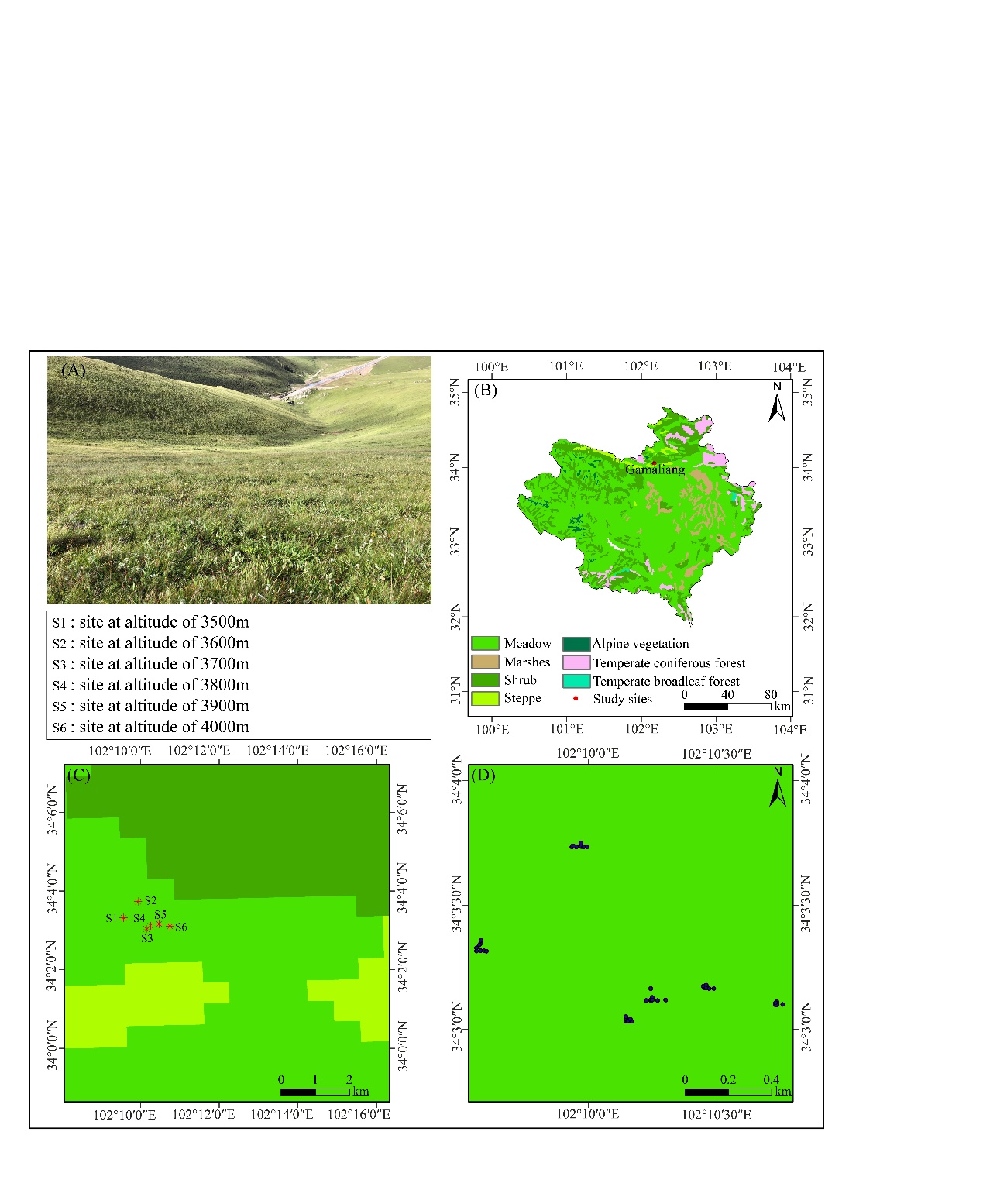
**

**Figure S1** Geographical location of Gamaliang and sampling sites in the Zoige Plateau


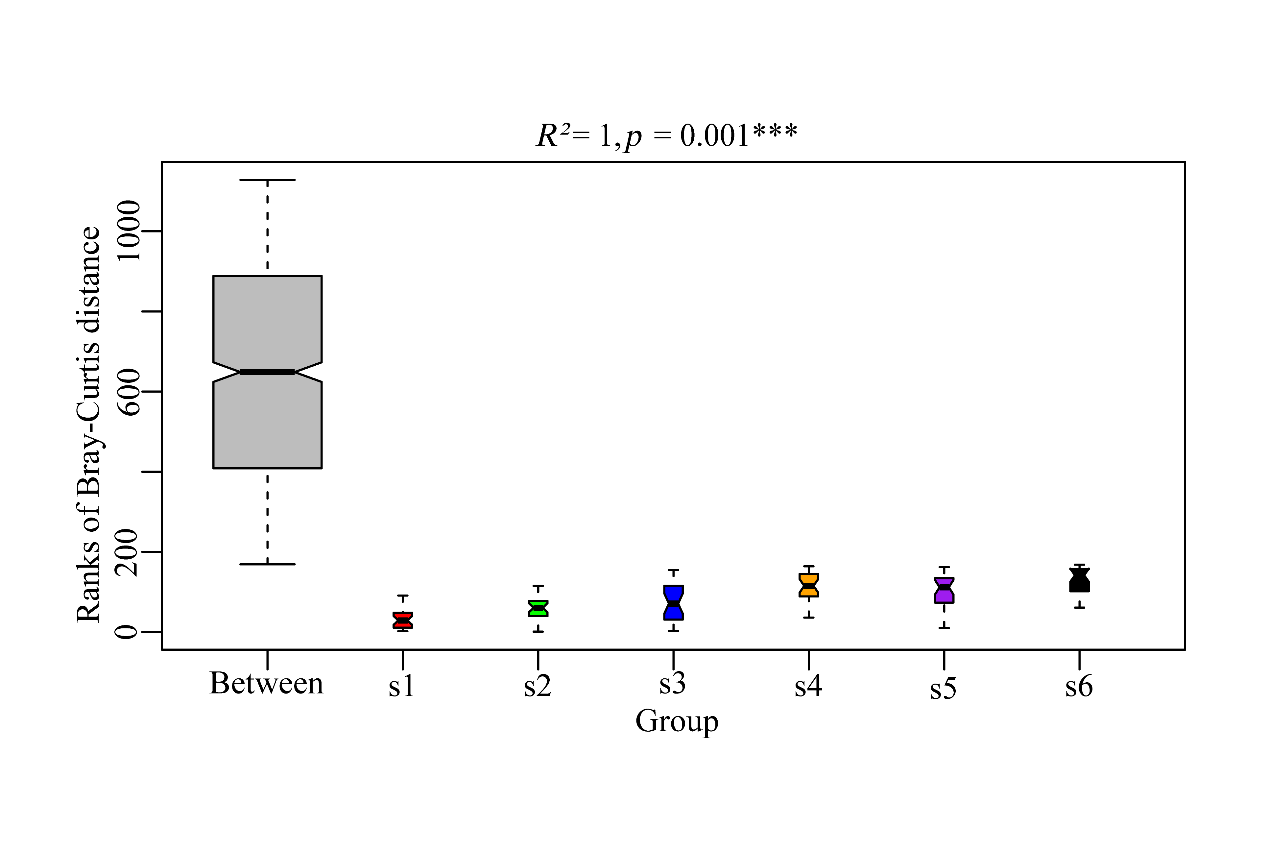


**Figure S2** Analysis of similarity (Anosim) of species assemblage similarity between altitude groups and within altitude groups


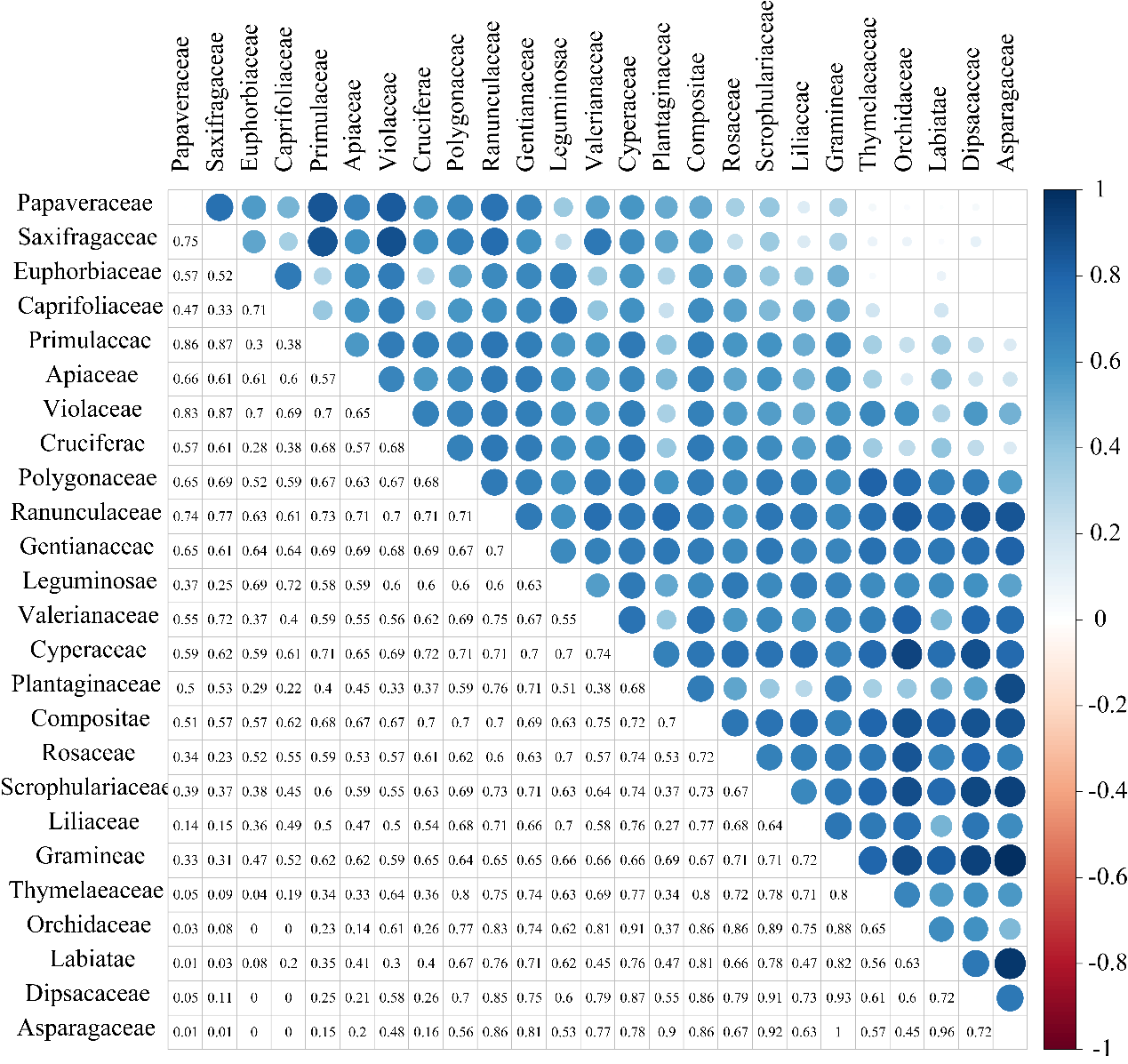


**Figure S3** Niche breadths and overlaps between plant families. Overlap values between two plant families were shown by the colors.

**Table S1** The Anosim and Adonis analysis of species assemblage similarity between random two altitude groups. Anosim, Analysis of similarity; Adonis, permutational multivariate analysis of variance; s1~s6 were sites at 3500~4000m altitude.

| group | Anosim | | Adonis | | |
| --- | --- | --- | --- | --- | --- |
|  | *R*^2^ | *p* | *F* | *R*^2^ | *p* |
| s1/s2 | 1 | 0.001**** | 24.18 | 0.63 | 0.001**** |
| s1/s3 | 1 | 0.002*** | 34.67 | 0.71 | 0.001**** |
| s1/s4 | 1 | 0.001**** | 63.09 | 0.82 | 0.002*** |
| s1/s5 | 1 | 0.001**** | 82.21 | 0.85 | 0.001**** |
| s1/s6 | 1 | 0.001**** | 114.98 | 0.89 | 0.001**** |
| s2/s3 | 1 | 0.001**** | 18.88 | 0.57 | 0.002*** |
| s2/s4 | 1 | 0.001**** | 40.09 | 0.74 | 0.001**** |
| s2/s5 | 1 | 0.001**** | 71.31 | 0.84 | 0.001**** |
| s2/s6 | 1 | 0.002*** | 89.26 | 0.86 | 0.001**** |
| s3/s4 | 1 | 0.001**** | 27.71 | 0.66 | 0.001**** |
| s3/s5 | 1 | 0.002*** | 47.94 | 0.77 | 0.001**** |
| s3/s6 | 1 | 0.002*** | 80.20 | 0.85 | 0.001**** |
| s4/s5 | 1 | 0.001**** | 36.84 | 0.72 | 0.001**** |
| s4/s6 | 1 | 0.001**** | 64.01 | 0.82 | 0.001**** |
| s5/s6 | 1 | 0.001**** | 60.86 | 0.81 | 0.001**** |

** p* <0.1, ***P*≤0.05, ****P*≤0.01, *****P*≤0.001.

**Table S2** Results of the selection of spatial eigenfunctions with positive eigenvalues based on the principal coordinates of neighbor matrices (PCNM).

| Sites | *Variable* | *F* | *p* | *R^2^* |
| --- | --- | --- | --- | --- |
| s1~s6 | PCNM1 | 17.09 | 0.001 | 0.255 |
|  | PCNM2 | 5.29 | 0.001 | 0.399 |
|  | PCNM3 | 7.02 | 0.001 | 0.341 |

**Table S3** Results of forward selection of environmental variables run by the ordiR2step function in the vegan package.

| Sites | Variable | *R^2^* | *F* | *p* |
| --- | --- | --- | --- | --- |
| s1~s6 | NO_3_^+^-N | 0.144 | 8.90 | 0.0002**** |
|  | SWC | 0.274 | 9.21 | 0.0001**** |
|  | AK | 0.354 | 6.58 | 0.0003**** |
|  | AP | 0.491 | 12.84 | 0.0001**** |
|  | TOC | 0.578 | 9.95 | 0.0001**** |
|  | PH | 0.646 | 9.03 | 0.0001**** |
|  | BK | 0.668 | 3.72 | 0.0030*** |
|  | TP | 0.683 | 2.85 | 0.0107** |

** p* <0.1, ***P*≤0.05, ****P*≤0.01, *****P*≤0.001.

**Table S4** The niche breadth of plant families. Mean.simulated, the mean value of zero distribution of the niche breadth index of a plant family; UppCI, the upper 95% confidence interval; LowCI, the lower 95% confidence interval; Sign, a plant family was considered a generalist or specialist based on whether the habitat niche breadth value exceeded UppCI or fell below LowCI; no significant, the observed value of habitat niche breadth was located between LowCI and UppCI.

| Family | Habitat niche breadth | Mean.simulated | LowCI | UppCI | Sign |
| --- | --- | --- | --- | --- | --- |
| Gramineae | 40.96 | 33.42 | 28.86 | 38.04 | generalist |
| Cyperaceae | 45.40 | 36.79 | 32.52 | 40.62 | generalist |
| Leguminosae | 40.85 | 32.84 | 28.35 | 36.96 | generalist |
| Compositae | 45.09 | 31.01 | 25.61 | 35.51 | generalist |
| Rosaceae | 38.90 | 32.81 | 28.31 | 37.00 | generalist |
| Ranunculaceae | 45.77 | 28.53 | 23.36 | 33.47 | generalist |
| Polygonaceae | 42.75 | 32.05 | 27.01 | 36.40 | generalist |
| Gentianaceae | 44.34 | 28.66 | 23.13 | 33.31 | generalist |
| Plantaginaceae | 22.22 | 23.56 | 18.30 | 28.66 | non significant |
| Scrophulariaceae | 38.50 | 29.19 | 24.36 | 34.16 | generalist |
| Labiatae | 23.23 | 24.08 | 18.60 | 29.14 | non significant |
| Liliaceae | 34.66 | 33.10 | 28.29 | 37.27 | non significant |
| Valerianaceae | 36.59 | 33.05 | 28.16 | 37.44 | non significant |
| Violaceae | 43.53 | 35.82 | 31.48 | 39.78 | generalist |
| Thymelaeaceae | 22.37 | 25.70 | 20.82 | 30.23 | non significant |
| Orchidaceae | 13.46 | 18.74 | 13.95 | 23.40 | specialist |
| Dipsacaceae | 14.11 | 19.59 | 14.56 | 24.03 | specialist |
| Asparagaceae | 8.67 | 15.19 | 10.90 | 19.88 | specialist |
| Apiaceae | 37.73 | 35.12 | 30.53 | 39.04 | non significant |
| Caprifoliaceae | 28.79 | 31.57 | 26.70 | 36.15 | non significant |
| Euphorbiaceae | 21.37 | 27.20 | 21.45 | 32.05 | specialist |
| Primulaceae | 42.63 | 33.25 | 28.59 | 37.83 | generalist |
| Cruciferae | 39.63 | 29.40 | 24.55 | 34.39 | generalist |
| Papaveraceae | 13.04 | 18.26 | 13.55 | 23.20 | specialist |
| Saxifragaceae | 9.33 | 14.90 | 10.96 | 18.94 | specialist |
